# The Importance of Selected Coordination Motor Skills for an Individual Football Player’s Effectiveness in a Game

**DOI:** 10.3390/ijerph19020728

**Published:** 2022-01-10

**Authors:** Łukasz Bojkowski, Paweł Kalinowski, Robert Śliwowski, Maciej Tomczak

**Affiliations:** 1 Department of Psychology, Poznan University of Physical Education, Królowej Jadwigi 27/39, 61-871 Poznań, Poland; mtomczak@awf.poznan.pl; 2 Department of Theory and Methodology of Team Sport Games, Poznan University of Physical Education, Królowej Jadwigi 27/39, 61-871 Poznań, Poland; pawel.kalinowski1030@gmail.com (P.K.); sliwowski@awf.poznan.pl (R.Ś.)

**Keywords:** football, coordination motor skills, offensive play, defensive play, comprehensive evaluation of the effectiveness of the game

## Abstract

The appropriate level of coordination motor skills (CMS) in a football player is one of the factors determining the effectiveness of their actions. Adaptability and complex reaction time are of particular importance in models of coordination requirements in football. The lead aim of this study is to determine the relationship between two selected coordination motor skills and the offensive, defensive and comprehensive effectiveness of an individual player’s actions. The study was conducted on a group of 91 Polish male football players aged 20 to 31 years, all in the senior age category. The research tools included: a test assessing motor adaptation (research by dribbling the ball with the dominant leg), psychomotor test of complex reaction time (tested with an S-10.2 measuring device) and a test of the effectiveness of an individual player’s actions (one-on-one simulation game). The conducted research indicated that adaptability and complex reaction time are both important abilities for success when attacking in an individual game, and in the assessment of a comprehensive index of individual competences in a one-on-one football game. However, the most significant factor influencing the effectiveness of a player’s defensive action is solely the complex reaction time.

## 1. Introduction

Football is one of the most popular sports disciplines in the world. The data reported on the website of the international football federation, FIFA (fr. Fédération Internationale de Football Association) indicates that, in 2009, over 265 million male and female players participated in various organized competitions around the world, and there are 211 associations affiliated to FIFA in 2021 [1]. Certain data indicate high competitiveness in football activities, and so, in order to achieve the best results, a player should have certain levels of key abilities, including technical, tactical, mental and motor skills. The latter includes, among others, coordination motor skills (CMS), which is the subject of this study.

When talking about effective performance in sport, an appropriate level of motor skills is high on the list [2,3]. These include coordination motor skills (CMS), which are one of the primary factors influencing a player’s effectiveness during a match [4,5,6]. These findings are supported by studies indicating that increasing the level of specific coordination motor skills is one of the most important factors in achieving the intended performance results [7]. As research explains [8], the most important criterion in sports, determining whether a player is picked to play or not, apart from the game evaluation, is the assessment of individual potential, in which the level of coordination motor skills plays a decisive role.

Coordination motor skills are determined by cognitive and control-regulatory processes, which means that a person’s coordination predisposition is determined by the system of regulation and control of movements—i.e., the efficiency of the information organization mechanism [7]—and their external expression is manifested in the economy and precision of movement. This makes it easier for players with a high level of specific abilities to learn new skills and motor activities, as they can rely on a wide range of technical [9] and motor skills acquired during coordination training at an earlier age [10]. This approach has already been adopted by national football federations, for example in Germany, where a coordination plan is one of the goals of the modern “DFB Talent Support Program” [11]. Studies have also indicated the existence of positive relationships between the coordination indices of motor skills and the level of general and special fitness [12].

Although the choice of the significance of individual coordination motor skills in a football game may differ—that is, the opinions of different researchers differ [7,13]—models of coordination requirements in football often emphasise the importance of two coordination motor skills:

1. Adaptability (motor adaptation)—this ability is defined as the ability to quickly adjust the developed forms of movements or to shift from one motor activity to another according to changing conditions [7], either existing or probable. Adaptability is responsible for 26.07% of the variance in the common structure of coordination abilities in male football players. It also correlates with the ability to combine movements (16.7%), rhythmisation ability (13.9%) and balance (9.3%) [7]. In sport science, motor tests of the same kind are often used as a research tool for assessing these coordinating motor skills. The test used in the conducted study involved running around multiple poles and dribbling the ball with the dominant leg [7];
2. Complex reaction time—this is defined as the ability to quickly perform a complete, short-term movement in response to a known or previously unknown signal (optical, acoustic, tactile, kinesthetic) with the whole body or a part of it [7]. Among the various forms of assessing a player’s reaction time, in football the focus is primarily on reacting quickly to a set of stimuli, which is expressed in the fact that, when a player is faced with a large number of different signals, they respond to each of them differently [7]. In football, as in other sports, the most commonly occurring quick reaction [7] is when a player must react in various ways to many signals, including distance from the goal, position on the pitch, or the movements of opponents and partners [7,14,15]. One of the research tools for assessing reaction time in athletes are measuring devices, and in the conducted study, an apparatus was used to test the respondent’s reaction time in response to a visual signal (typical for sports rivalry in football).

Although football is a team game, it often consists of one-on-one duels, which, in Szwarc’s definition [16], is the individual action of a player with the ball against a single opponent or the actions of one player without a ball against an opponent with the ball in situations of relative independence from teammates. Due to the nature of these actions, individual roles in duels are divided into offensive and defensive. When attacking in an individual duel, the player with the ball primarily aims to perform actions that will enable him to freely carry out the tasks of the game and move past the opponent (a player from the opposing team) in order to create a scoring chance or help to score, or with the intention of controlling the game and/or maintaining possession of the ball. In defending, by contrast, the player aims at the highest possible effectiveness of tasks that allow him to gain possession, clear the ball or make it difficult for the opponent to move. According to football coaches, winning such duels are one of the most important elements ensuring success in football [17,18,19] (the ability of a player to win duels is part of the standard model of assessing football talent [11]). In our study, the effectiveness of individual play (offense, defence and comprehensive) is tested through simulated one-on-one test games.

Taking into account the theoretical premises listed above, and assuming the significance of the two above-mentioned coordination motor skills for the effectiveness of a football player during a one-on-one game, the lead aim of our research is to determine the relationship between the selected coordination motor skills (adaptability, and complex reaction time) and the offensive, defensive and comprehensive effectiveness of the actions of individual footballers. The practical purpose of the study is to expand the coaching knowledge in the field, in particular the theory of training in a specific discipline, in this case football.

## 2. Materials and Methods

### 2.1. Participants

The observational study participants included players representing Polish football clubs, playing during the 2015/2016 season in league games organized by the Wielkopolska Football Association (WZPN) at five league levels:

1. III league (N = 28)—players aged 20 to 28 (M = 22.8, Me = 22, SD = 1.93), with training experience from 6 to 15 years (M = 11, Me = 11.5, SD = 2.65).
2. IV league, North Division (N = 35)—players aged 20 to 27 (M = 22.5, Me = 22, SD = 1.7), with training experience from 5 to 15 years (M = 9.8, Me = 9, SD = 2.55).
3. Regional league, East Division (N = 11)—players aged 20 to 31 (M = 23.5, Me = 22, SD = 3.7), with training experience from 6 to 17 years (M = 10.9, Me = 11, SD = 3.56).
4. A-class, III Division (N = 11)—players aged 21 to 30 (M = 24.3, Me = 24, SD = 2.97), playing a specific sport from 6 to 18 years (M = 10.8, Me = 11, SD = 3.76).
5. B-class, II Division (N = 6)—players aged 20 to 23 (M = 21.7, Me = 22, SD = 1.5), playing football from 7 to 15 years on average (M = 9.2, Me = 8.5, SD = 3.76).

The total research group consisted of 91 male football players aged 20 to 31 (M = 22.9, Me = 22, SD = 2.3), with an average football training experience of 10.4 years (Me = 10, SD = 2.91). All subjects were assigned to 1 of the 13 test subgroups (7 players in each). Each subgroup consisted of players from all the above-mentioned playing levels.

The players were tested from May to August 2015 on training pitches with artificial turf located in the immediate vicinity of the Municipal Stadium in Poznań (Greater Poland Voivodeship; Poland). All research was conducted in the direct presence of the lead author of the article. All players were tested on so-called non-training days in very similar weather conditions, and with the same basic and organizational preparation. In our research, the criterion for including and excluding players from the study was: having a minimum of five years’ training experience, representing their club in national competitions, and participation in training and sports competition in the senior age category. The study was approved by the Research Ethics Committee of the Karol Marcinkowski University of Medical Science in Poznań (approval number 780/14; date: 2 October 2014).

### 2.2. Measurement Tools

The choice of research tools was dictated by the conceptualisation of the research, in which a player’s effectiveness in a one-on-one football game is determined by the level of specific motor skills. On this basis, specific research variables (described above) were extracted, and a series of research tests was presented:

1. A test assessing the adaptability (motor adaptation)—running around poles and dribbling the ball with the dominant leg [7].

This test was performed first on an artificial turf pitch in the direct presence of the tester. The main task of the tested person was to cover the designated distance as quickly as possible, running around three poles one after another, dribbling the ball with the dominant leg (with any part of the foot). Time measurement started from the moment the player started (on the signal of the examiner), that is, when his body crossed the start line, and stopped when the subject, after covering the distance, crossed the finish line with any part of his body (from the neckline to the waist) [7]. The results were measured with an accuracy of 0.01 s. The study focused on controlling the ball with the dominant limb, because it is used in-game and provides the player with precision and confidence during the game (which applies not only to football players) [15,20,21].

This is a standardised test used for group and individual assessments and is characterised by a reliability coefficient level of r = 63–81 [7].

2. A test assessing complex reaction time—tested with an S-10.2 measuring device.

This test was carried out on the next training day, before attempting the one-on-one test games. The examination was carried out only in the presence of the investigator conducting it the test (so as not to distract the participants). During the test, the test subjects were exposed, in randomised order, to two visual stimuli: a yellow light and a green light. In total, the subject had to demonstrate a response to 16 stimuli (8 yellow and 8 green) by holding appropriate triggers in each hand. The subject’s task in the test was to react as quickly as possible to the visual stimuli by extinguishing the lit light with their thumb (the order in which a specific colour appeared was not known to the examined person). The trigger for the right limb controlled the green light, while the trigger for the left turned off the yellow light. Before the test, the subjects were instructed to perform the tasks as quickly and accurately as possible.

3. A test simulation game that determined the player’s offensive, defensive and comprehensive effectiveness—a one-on-one test game, using two goals, and without goalkeepers [7].

After completing the complex reaction time test (as described above), the competitors participated in a one-on-one test game. The playing field consisted of a square measuring 20 × 20 m, divided into two halves (the square was marked with a different colour). Net goals (1.5 m wide × 1 m high) were placed on the goal lines. The players in each sub-group played a “round robin” tournament (each examined subgroup consisted of seven people, thus each player played six matches). The number of games depended on the size of the research group and was calculated using the formula: x = N (N − 1)/2 [7]. One match lasted 2 minutes followed by a 5–8 min break for passive or active rest. For each foul, a penalty kick was awarded, with an empty goal from the centre of the pitch. The use of hands when defending was not allowed [7]. An assistant referee stopped the playing time when the ball went out of play [7].

When assessing individual attacking effectiveness, an indicator of comprehensive effectiveness was used, in this case, a numerically higher result (more goals scored by a player during a one-on-one game) was treated as a better result (determining greater effectiveness). By contrast, for the assessment of individual defensive effectiveness, the numerically lower result (lower number of goals conceded during a one-on-one game) was considered the better one. Detailed guidelines on the organisation of the study using the same method can be found directly in the work of Ljach and Witkowski [7] and indirectly in the paper of Witkowski and Duda [22].

The testing method is a standardised way of quantifying the player’s actions during a sports game [7]. The reliability indicators developed to assess the effectiveness of one-on-one play in football are medium and high, and amount to 0.67 for the number of goals conceded (defence), 0.86 for the number of goals scored (attack) and 0.89 for goal difference (all results were statistically significant at the level of *p* < 0.05) [22].

### 2.3. Statistical Analysis

Pearson’s r correlation coefficient was used to assess the strength and direction of the relationship between subjective properties and effectiveness. In order to extract the set of the best predictors, the forward selection regression method was used [23]. A summary of the regression for dependent variables is presented in this paper, i.e., the assessment of individual effectiveness in offensive and defensive play, and comprehensive effectiveness in a one-on-one game, which is reflected in the predisposition to compete one-on-one. There are two potential predictors in the analysis. It is often assumed that there should be at least 10–15 observations per predictor [24]. Statistica 13.1 software (TIBCO Software Inc., Texas, TX, USA) was used for analysis.

## 3. Results

First, the analysis comprised of the relationship between adaptability and complex reaction time, and the above-mentioned indicator used to assess the effectiveness of offensive actions in a one-on-one football game (Table 1).

There were statistically significant negative correlations between adaptability and both individual attacking effectiveness and comprehensive effectiveness in a one-on-one football game. In addition, statistically significant negative relationships were noted between the level of complex reaction time and individual attacking effectiveness and comprehensive effectiveness in a one-to-one game, as well as a significant relationship between complex reaction time and individual effectiveness when defending (Table 1).This means that the better results a player obtains in the reaction speed test, the more shots they will attempt (offensive effectiveness) and the fewer goals they will concede (defensive effectiveness) in a test game, and thus a player’s overall effectiveness increases.

The properties indicated in Table 2 explain a total of 16% of the variance (the adjusted coefficient indicates 14%) of the presented level of the player’s effectiveness when attacking (F(2.88) = 8.376; *p* < 0.001). Adaptability, introduced in the first step, explained 12% of the variance, while complex reaction time in the second step increased the explanation of variance by 4%. In the obtained final model (two variables), a negative relationship between adaptability (Beta = −0.28; *p* < 0.01) and complex reaction time (Beta = −0.21; *p* < 0.05) was observed in regard to the player’s offensive effectiveness.

Complex reaction time, introduced in the first step, which explains 10% of the variance in effectiveness (according to the adjusted coefficient of 9%) (F(1.89) = 9.876; *p* < 0.01) (Table 3), turned out to be the only significant predictor of the presented level of a player’s effectiveness when defending.

The two variables described account for a total of 17.1% of its variance (the adjusted coefficient is 15.2%) (F(2.88) = 9.087; *p* < 0.001) of the level of a player’s comprehensive effectiveness in a one-on-one football game (Table 4). Complex reaction time introduced in the first step explained 12.8% of the variance, while adaptability added in the second step increased the explanation of variance by 4.28%. A key observation from the obtained final model (two variables) was a negative relationship between complex reaction time (Beta = −0.29; *p* < 0.01) and adaptability (Beta = −0.22; *p* < 0.05), and the player’s comprehensive effectiveness in a one-on-one football game.

## 4. Discussion

It has previously been determined that, for effective performance in a football match, as in other sports, the appropriate level of motor skills is of fundamental importance [3,5,6], including, in particular, coordination motor skills [25], which can determine a player’s competitive level [4]. High competence in this area may determine the quality of the tasks performed, especially when the players receive a substantial amount of information (internal and external), and their effective processing determines the effective performance of technical and tactical activities [26,27]. The existence of a large number of studies, focused on various levels of competition, testifies to the usefulness of undertaking such analyses [28,29,30].

The results of linear correlation and multiple regression studies indicate that the most important coordination motor skills influencing individual success when attacking are both adaptability and complex reaction time (the former explains the largest percentage of variance). In the case of the effectiveness of individual defensive play, however, the most important factor among the studied variables (coordination motor skills) is complex reaction time.

The developed model for the comprehensive effectiveness of an individual game in football explains about 15% of the variance, the model for the effectiveness of the game in attack explains about 14% of the variance, and the model for the effectiveness of the game in defence about 9% of the variance. These values are relatively small, which indicates the need for further searching of the variables that are important for the effectiveness of football players. At the same time, it should be noted that, in the conducted analysis, only two motor coordination abilities (adaptability and complex reaction time) were examined, hence it is difficult to expect that two variables from one area would sufficiently explain as complex a phenomenon as the effectiveness of action in football.

In explaining the obtained results, it is worth considering the range of actions that attacking players are required to perform. They must adapt and react to the actions of the opposing team’s defenders and their own teammates, as well as make quick decisions concerning actions characterised by their high level of creativity during the implementation of individual solutions (using dribbling or dummies). Furthermore, the characteristics of an offensive player are also based on quick reactions to many signals, such as distance from the goal, opponents’ movements [14,15]. By contrast, a defensive player’s main task is to react quickly to situations created by their opponents, including blocking shots or anticipating their opponents’ actions to enable them to receive or knock the ball away [17,18,19]. Thus, adaptability during the game of football is manifested, among others, in such individual activities as: improvisation in the performance of dummies during a one-on-one game; change in the performance of special tasks; the manner of receiving, dribbling, kicking the ball or using dummies in response to the conditions and a player’s position on the pitch; playing against different opponents; playing in different tactical positions and systems; adapting to the game in cooperation with different and/or new partners; playing on different pitches; combining movements; and improvising in unusual situations during the game [7,31]. On the other hand, complex reaction time manifests itself in such situations as: immediate defensive action, passing the ball to a moving teammate, run to the ball, an immediate shot on goal, reacting to shots on goal, clearing the ball, receiving the ball, [7] and when it is necessary, responding quickly to changing game conditions.

The obtained results also indicate the importance of coordination motor skills, such as adaptability and complex reaction time, in the assessment of a comprehensive index of action competences in a one-on-one game, which has been described by researchers as the most difficult skill in football [7,16].This specific result can be explained by the fact that a high level of adaptability, as well as the ability to react quickly is necessary for adequate functioning in complex and highly variable sports conditions, including football, which is characterised by a range of features including: high dynamics and constant situational variability, fast pace of the game and the change of its rhythm [3], when considering not only offensive and defensive phases, but also activities typical of transitional phases. What is more, based on the tested coordination abilities, new skills are often acquired, or existing skills are developed and improved [32]. It influences the assessment of the quality of mastering and performing difficult and temporally and spatially complex activities based on the typical technique for a given sport [33], and also helps to meet the increasingly higher requirements concerning psychophysical traits [34].

The conclusions obtained on the basis of our research are largely consistent with research that has observed that some of the basic factors determining the efficiency of the individual actions of a player in team sports are, among others, motor skills, and cognitive and decision-making factors, as well as conditioning and coordination abilities. The justification for the results can also be obtained in the literature on the subject, where it has been stated that the ability to react quickly and effectively to a ball travelling at high speed is an important factor influencing the efficiency of an action in football [35].This becomes significant when one takes into consideration the fact that, in comparison to all other sports [10], football requires the highest level of coordination; it is one in which accuracy, speed—or speed adjustment—and variability are expected, i.e., the spatial accuracy of movements performed in minimum units of time under changing conditions of competition [36].

In the case of motor properties, both a high level of adaptability, as well as quick complex reaction time are important for the performance level of a football player in a one-on-one game. Therefore, it is recommended that coaching staff support the development of specific coordination motor skills [7,29], as well as control the changes taking place due to the influence of the training loads employed [37,38].

The limitation of our research is the fact that certain results were obtained only on a group of senior male players training and competing in football competitions. One interesting avenue of research would be to conduct similar studies in other age categories, e.g., in juniors, and in women. The second limitation is the analysis of only motor skills, including two selected coordination motor skills—adaptability and complex reaction time (their selection was justified in the relevant section). Introducing additional features of players would certainly expand the topic under discussion.

## 5. Conclusions

On the basis of the conducted study, the following specific final conclusions can be specified:

1. The level of adaptability and complex reaction time are important for the individual effectiveness of a one-on-one game of football in attack, and complex reaction time for individual effectiveness of the game in defence;
2. The level of coordination motor skills, such as adaptability and complex reaction time, is related to the level of a player’s comprehensive effectiveness in a one-on-one football game;
3. Future research directions may be concerned with the replication of these parameters on more closely selected groups (e.g., national team players) or/and introducing additional psychomotor variables.

## Figures and Tables

**Table 1:** Relationships between the levels of the studied coordination motor skills and the indicators of the assessment of an individual player’s effectiveness in football—Pearson’s r correlation coefficients.

Variables	N = 91	
	AD	CRT
AT	r = −0.346; *p* = 0.001	r = −0.304; *p* = 0.003
DEF	r = 0.195; *p* = 0.064	r = 0.316; *p* = 0.002
COMP	r = −0.314; *p* = 0.002	r = −0.358; *p* = 0.000

AD—adaptability; CRT—complex reaction time; AT—effectiveness in attack; DEF—effectiveness when defending; COMP—comprehensive game effectiveness.

**Table 2:** Multiple regression—predictors of game effectiveness in attack.

N = 91	R = 0.400; R^2^ = 0.160; Adjusted: R^2^ = 0.141 F(2.88) = 8.376; *p* < 0.000; SE of Estimate: 3.896					
	b *	SE of b *	b	SE of b	t(88)	*p*
Intercept			28.746	4.988	5.763	0.00000
AD	−0.276	0.104	−1.401	0.526	−2.662	0.00922
CRT	−0.213	0.104	−0.236	0.115	−2.053	0.04307

AD—adaptability; CRT—complex reaction time; *—standardized coefficient.

**Table 3:** Multiple regression—predictors of effectiveness when defending.

N = 91	R = 0.316; R^2^ = 0.099; Adjusted: R^2^ = 0.089 F(1.89) = 9.876; *p* < 0.002; SE of Estimate: 3.984					
	b *	SE of b *	b	SE of b	t(89)	*p*
Intercept			−0.763	3.114	−0.245	0.80708
CRT	0.316	0.101	0.348	0.112	3.143	0.00228

CRT—complex reaction time; *—standardized coefficient.

**Table 4:** Multiple regression—predictors of comprehensive game effectiveness.

N = 91	R = 0.414; R^2^ = 0.171; Adjusted: R^2^ = 0.152 F(2.88) = 9.087; *p* < 0.000; SE of Estimate: 6.710					
	b *	SE of b *	b	SE of b	t(87)	*p*
Intercept			33.595	8.592	3.910	0.00018
CRT	−0.286	0.103	−0.549	0.198	−2.778	0.00668
AD	−0.220	0.103	−1.932	0.906	−2.132	0.03578

CRT—complex reaction time; AD—adaptability; *—standardized coefficient.

## Data Availability

The data presented in this study are available on request from the corresponding author.
